# Supplementary material for: Beyond venomous fangs: Uloboridae spiders have lost their venom but not their toxicity
Source: BMC Biol. 2025 Jun 13;23:159. doi: 10.1186/s12915-025-02248-1 (PMC12164160; doi:10.1186/s12915-025-02248-1)
Supplement: Supplementary file 1 — Additional file 1. Tables S1–S4 and Figures S1–S7. Table S1 RNA-seq library summary statistics. Table S2 De novo transcriptome assembly summary statistics. Table S3 List of the 11 spider genomes used for the assembly annotation. Table S4 List of fastq files used to verify and quantify defensin in the genome of Octonoba sinensis. Fig. S1 OMArk completeness evaluation of U. plumipes transcriptome assembly. Fig. S2 Violine plot of library transcripts per million (TPMs) data. Fig. S3 Read count density of libraries. Fig. S4 Principal component analysis using the top 1000 most variable transcripts. Fig. S5 Top defensin blast-hit regions of U. plumipes genome. Fig. S6 Expression levels of the newly annotated defensin genes in the Octonoba sinensis genome. Fig. S7 Alignment of predicted neurotoxins with cysteine frame highlighted. [file 12915_2025_2248_MOESM1_ESM.pdf]

Table S1: RNA-seq library summary statistics. UC1-3: chelicerae; UP1-3: prosoma; UG: gonads; UM1-3: midgut glad; US1-3: silk glands.

Libraries	Total raw reads	High quality reads
**UC1**	58'494'780	48'903'542
**UC2**	53'421'906	38'774'986
**UC3**	54'406'220	38'713'640
**UP1**	55'299'872	46'506'656
**UP2**	75'303'620	67'291'910
**UP3**	98'080'052	74'005'606
**UG1**	107'756'820	96'386'224
**UM1**	96'262'266	85'748'874
**UM2**	90'920'880	83'279'616
**UM3**	95'046'808	86'684'478
**US1**	53'881'834	44'579'252
**US2**	53'881'834	48'642'782
**US3**	53'941'648	49'819'522

Table S2: *De novo* transcriptome assembly summary statistics. ORF: open reading frame.

	# Transcripts	
	**Complete ORF**	**Incomplete ORF**
**Assembled sequences**	302'504	
**Transcripts with ORF**	159'668	66'125
**Annotated sequences**	33'129	260
**Clustered sequences***	20'050	193

* After grouping sequences with 99% identity.

Table S3: List of the 11 spider genomes used for the assembly annotation.

Assembly accession	Species	Total sequence length	Assembly level	Assembly submission date
**GCA_013235015.1**	*Araneus ventricosus*	3'656'621'265	Scaffold	2019-08-02
**GCA_015342795.1**	*Argiope bruennichi*	1'670'285'661	Chromosome	2020-11-16
**GCA_021605075.1**	*Caerostris darwini*	1'501'919'382	Scaffold	2021-11-19
**GCA_021605095.1**	*Caerostris extrusa*	1'420'656'204	Scaffold	2021-11-19
**GCA_019974015.1**	*Nephila pilipes*	2'694'500'076	Scaffold	2021-07-23
**GCA_019343175.1**	*Oedothorax gibbosus*	821'427'276	Chromosome	2021-08-05
**GCA_000365465.3**	*Parasteatoda tepidariorum*	1'228'972'128	Scaffold	2019-06-14
**GCA_010614865.2**	*Stegodyphus dumicola*	2'551'176'228	Scaffold	2020-02-14
**GCA_019973975.1**	*Trichonephila clavata*	2'497'895'991	Scaffold	2021-07-23
**GCA_019973935.1**	*Trichonephila clavipes*	2'874'350'602	Scaffold	2021-07-23
**GCA_019973955.1**	*Trichonephila inaurata madagascariensis*	2'507'041'000	Scaffold	2021-07-22

Table S4: List of fastq files used to verify and quantify defensin in the genome of *Octonoba sinensis*.

**SRA**	**Species**	**tissue**
SRR26131816	*Octonoba sinensis*	Brain
SRR26148751	*Octonoba sinensis*	Abdomen
SRR26148754	*Octonoba sinensis*	Brain
SRR26148755	*Octonoba sinensis*	Brain
SRR26148758	*Octonoba sinensis*	Chelicera
SRR26148759	*Octonoba sinensis*	Chelicera
SRR26148770	*Octonoba sinensis*	Silk gland
SRR26148771	*Octonoba sinensis*	Silk gland
SRR26148772	*Octonoba sinensis*	Silk gland
SRR26148773	*Octonoba sinensis*	Gut
SRR26148774	*Octonoba sinensis*	Gut
SRR26148775	*Octonoba sinensis*	Gut
SRR26148776	*Octonoba sinensis*	Abdomen
SRR26148777	*Octonoba sinensis*	Abdomen

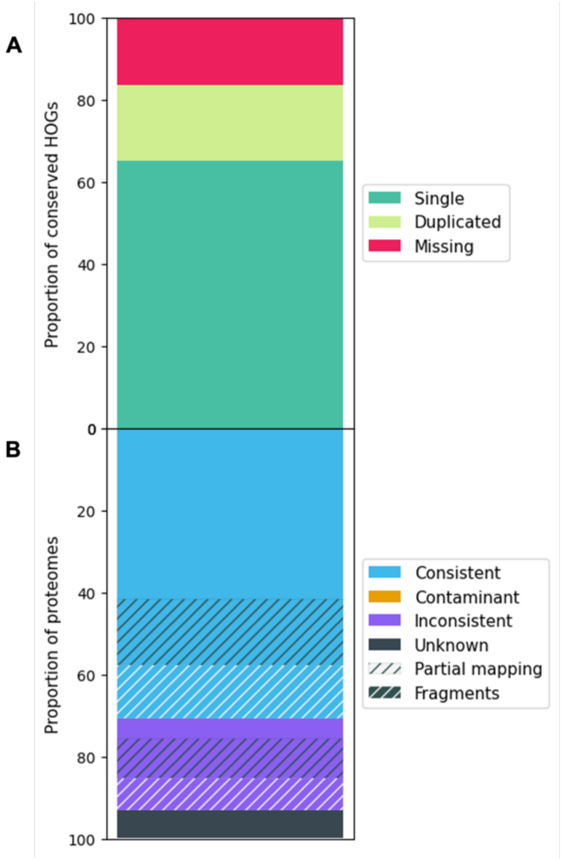

Fig. S1 OMArk completeness evaluation of *U. plumipes* transcriptome assembly. **A**. Proportion of conserved Hierarchical Ortholog groups (HOGs) using Arthropoda as ancestral clade which contains 3’589 conserved HOGs. Results on conserved HOGs: Single: 2’335 (65.06%); Duplicated: 662 (18.45%); Duplicated, Unexpected: 639 (17.80%); Duplicated, Expected: 23 (0.64%); Missing: 592 (16.49%). **B**. Proportion of proteins in the transcriptome with a consistent lineage placement: Total consistent, 14’328 (70.78%); Consistent, partial hits, 3’290 (16.25%); Consistent, fragmented: 2’617 (12.93%). Inconsistent lineage placements: Total inconsistent, 4’512 (22.29%); Inconsistent, partial hits: 1’948 (9.62%); Inconsistent, fragmented: 1’547 (7.64%). Total unknown: 1’403 (6.93%).

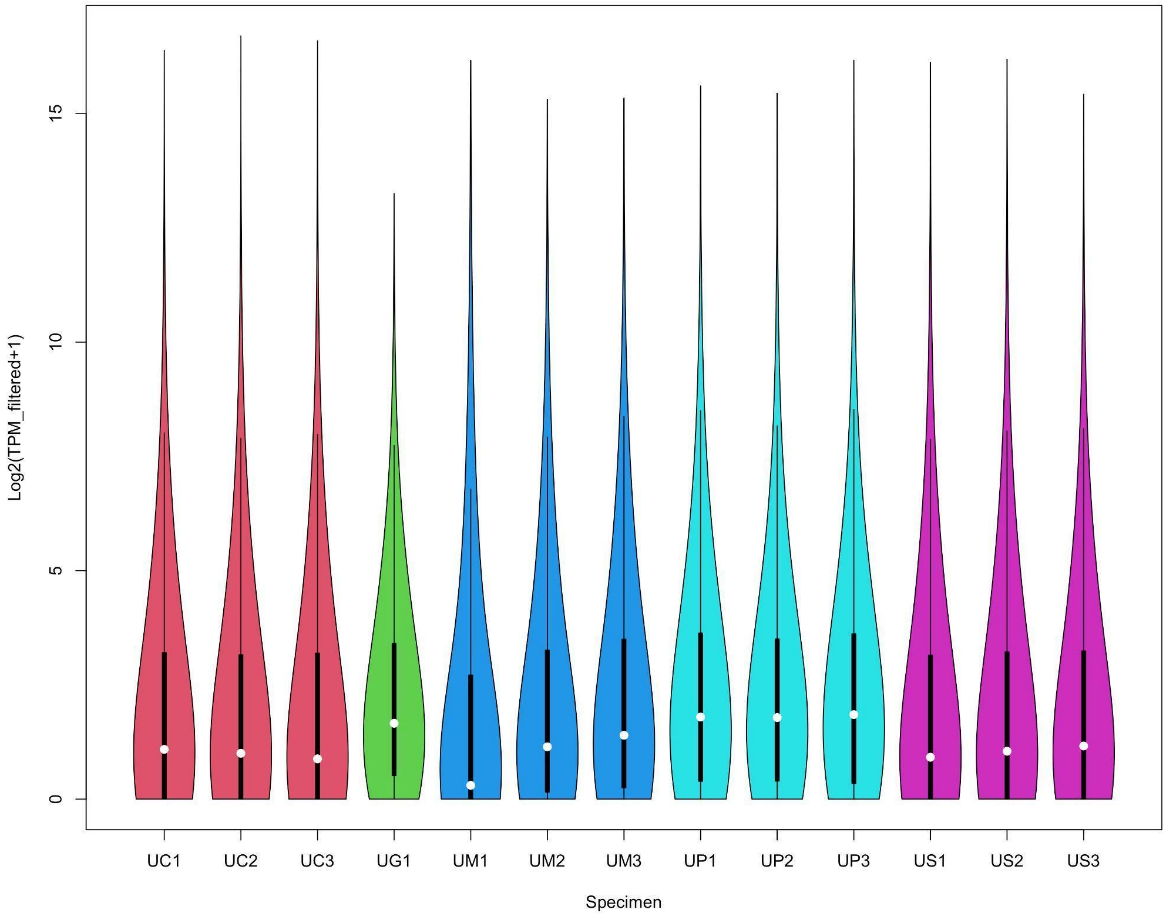

Fig. S2 Violine plot of library transcripts per million (TPMs) data. UC1, UC2, UC3: chelicerae; UG1: gonad; UM1, UM2, UM3: midgut gland; UP1, UP2, UP3: prosoma; US1, US2, US3: silk glands.

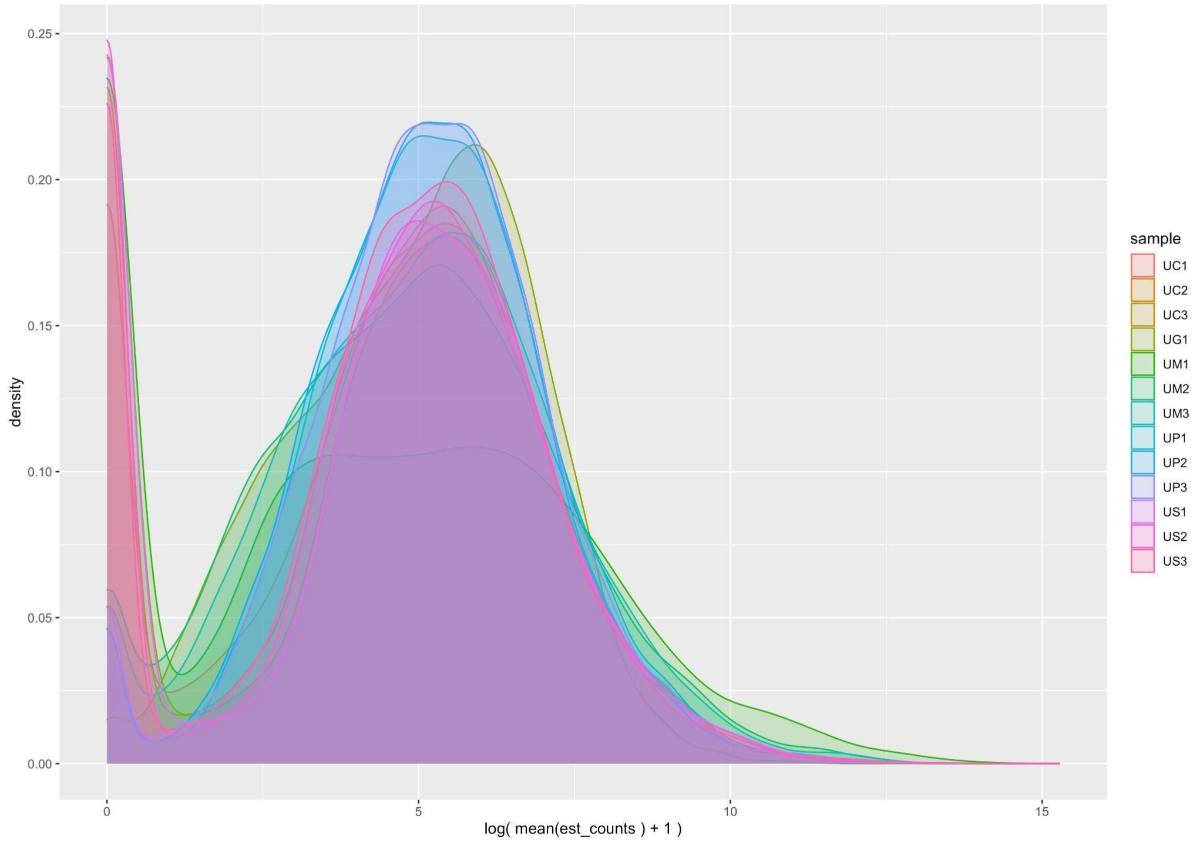

Fig. S3 Read count density of libraries. UC1, UC2, UC3: chelicerae; UG1: gonad; UM1, UM2, UM3: midgut gland; UP1, UP2, UP3: prosoma; US1, US2, US3: silk glands.

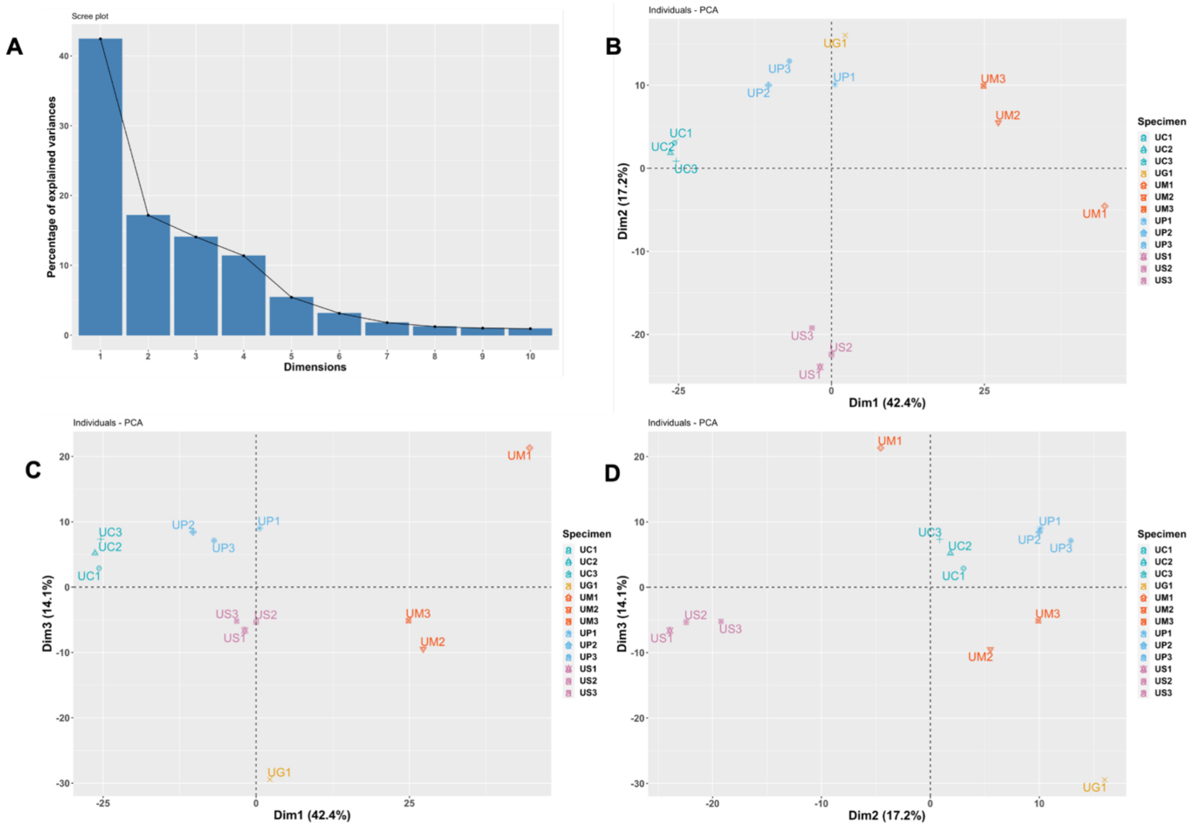

Fig. S4 Principal component analysis using the top 1’000 most variable transcripts. A. Scree plot of the components’ explained variance. **B, C, D**. PCA plot with the first two component (**B**), the first and third (**C**), and second and third components (**D**). UC1, UC2, UC3: chelicerae; UG1: gonad; UM1, UM2, UM3: midgut gland; UP1, UP2, UP3: prosoma; US1, US2, US3: silk glands.

A

B

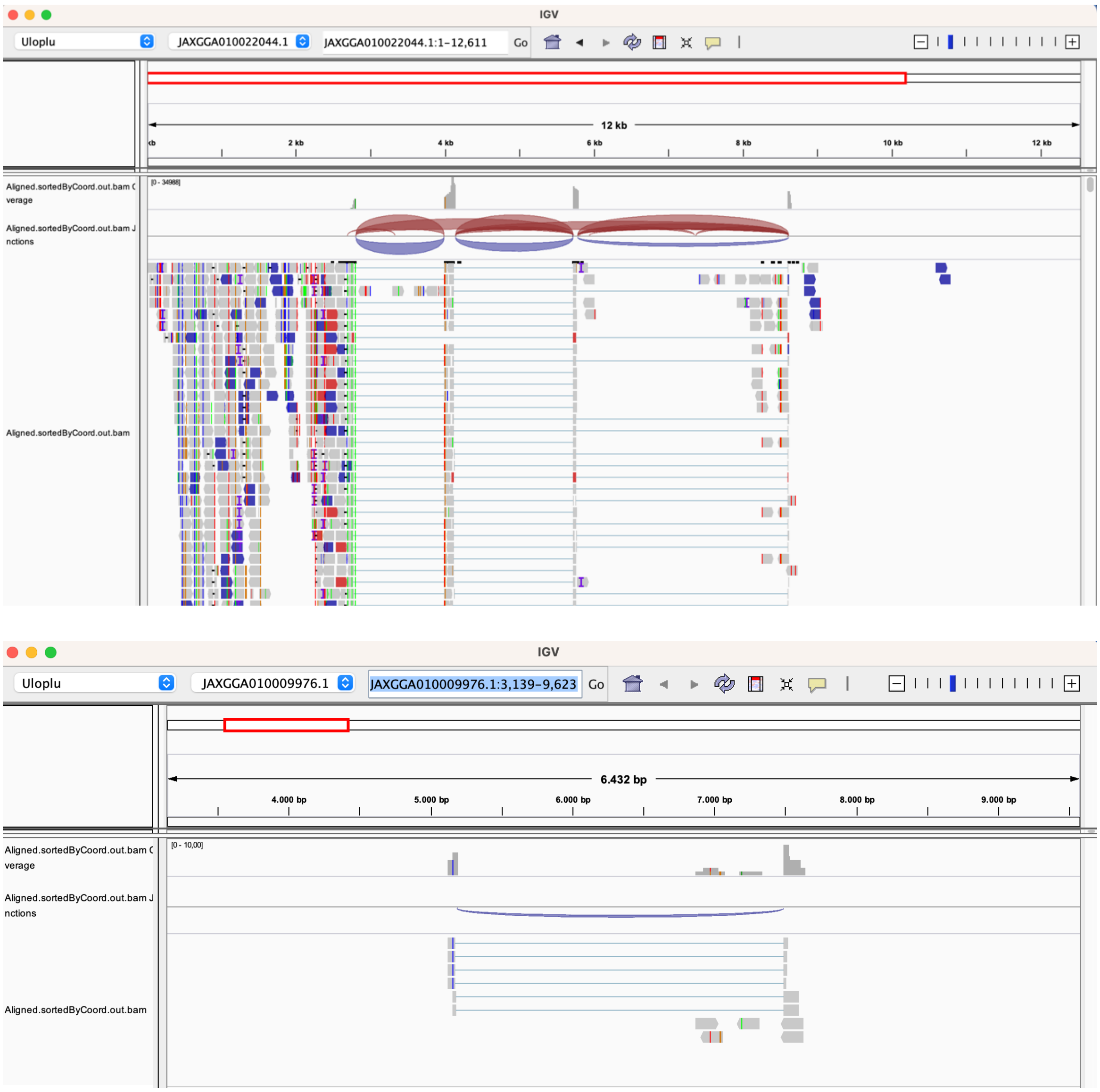

Fig. S5 Top defensin blast-hit regions of *U. plumipes* genome. A. Most RNA-Seq reads align to the top BlastN hit, which was 100% identical to our defensin transcript. **B.** Very few reads map to the second BlastN hit.

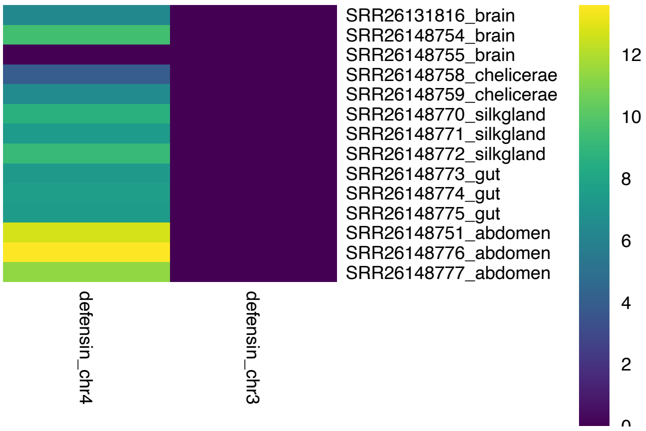

Fig. S6 Expression levels of the newly annotated defensin genes in the *Octonoba sinensis* genome. Expression levels as $\log_2$ (TPM).

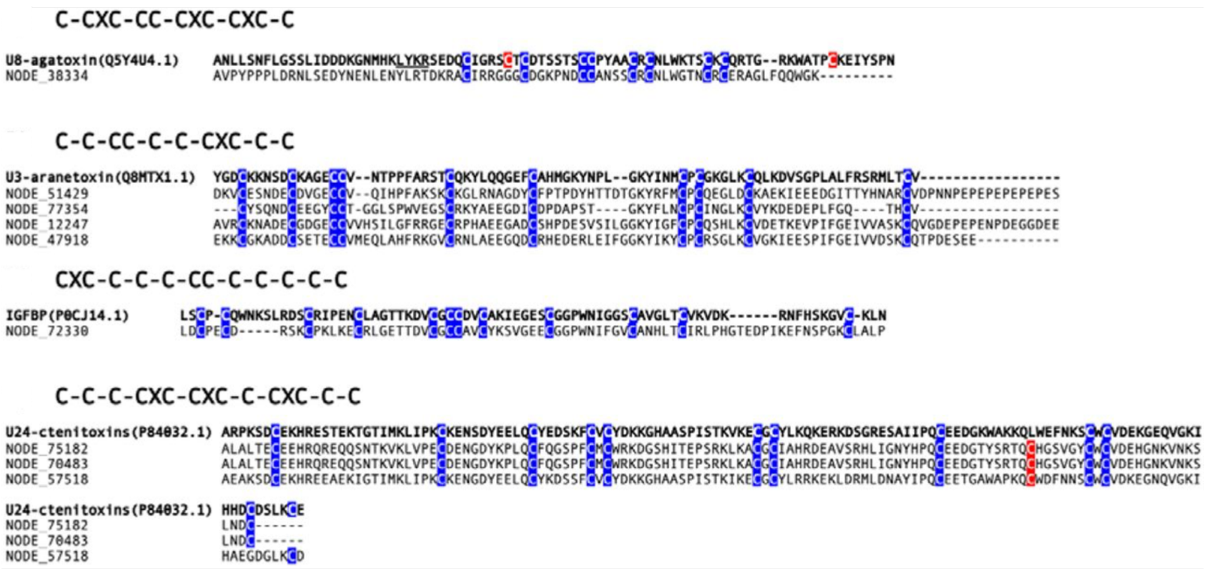

Fig. S7 Alignment of predicted neurotoxins from the *U. plumipes* transcriptome. The cysteine frame is reported.
